# eHealth, teledentistry and health workforce challenges: results of a pilot project

**DOI:** 10.1186/s12903-022-02603-6

**Published:** 2022-12-01

**Authors:** Orsolya Németh, Fanni Simon, Abdallah Benhamida, Márton Kivovics, Péter Gaál

**Affiliations:** 1 Department of Community Dentistry, Faculty of Dentistry, Semmelweis University, Budapest, 1088 Hungary; 2 Health Services Management Training Centre, Faculty of Health and Public Administration, Semmelweis University, Budapest, 1125 Hungary; 3 Department of Applied Social Sciences, Faculty of Technical and Human Sciences, Târgu-Mureș, Hungarian University of Transylvania, Transylvania, Romania; 4 Abdallah Benhamida BioTech Research Center, Óbuda University, Bécsi Út 96/B, Budapest, 1034 Hungary

**Keywords:** Public health, Epidemiology, Health workforce, eHealth, Telecare, Telemedicine, Teledentistry, Dental health education

## Abstract

**Background:**

In the twenty-first century, health systems have to cope with the challenges posed by their rapidly changing environment. Among these changes, the emergence of digital health solutions is an opportunity to make health systems better, but also a compelling force to change. Community dentistry is one area of health care, where the rapid technological development has the potential for substantial performance improvement benefitting dental patients in terms of access to care and conveniance.

**Methods:**

This study is based on a survey of the dental care provided by three units (Oral Medicine, Periodontology, Orthodontics) of the Department of Community Dentistry, Semmelweis University, Budapest. During a period of 12 weeks, we have collected time balance data on 1131 patients, 539 in the traditional and 592 in a pilot teledentistry setting, in order to estimate how much time could be spared by monitoring patients through videoconferencing instead of face-to-face visits.

**Results:**

According to our findings, teledentistry has the potential to shorten the visit with an average of 5–10 min per patient, which adds up to 58–116 work hours in a year. If the pilot was rolled out to all the 13 chairs of the surveyed 3 specialties (orthodontics, periodontology and oral medicine) the time saving would sum up to 186 workdays in one shift alone, which would translate to close to 4500 additional patients per year, considering remote patient monitoring cases alone. Further, if inactive doctors and highly qualified dental hygienists were involved in delivering telecare, 2.67 times as many workdays could be spared, which would allow about 12,000 more patients treated per year.

**Conclusions:**

The rapid development of digital health technologies coupled with the evolving task distribution between health professionals have a great potential to improve health system performance in pursuit of population health. Unfortunately, the adaptation to these technological changes is uneven, and without a national strategy, the poor will unlikely benefit from these opportunities in public dental care.

## Background

Health systems of the twenty-first century have to cope with the challenges posed by their rapidly changing socioeconomic, ecological, technological, demographic and epidemiological environment. The revolution of information and communication technologies (ICT) has the potential to make contemporary health systems more effective, efficient and equitable at the same time, but represents an external challenge on their own right, as well, which health systems have to adapt to. The use of digital health solutions, therefore, is not just an opportunity to make health systems better, but a compelling force to change, which disrupts the traditional organizational and operational framework of medical care.

Digital health solutions, such as eHealth, mHealth, Big and Long Data, are ICT based medical and management technologies to improve the performance of health systems [1–5]. The ICT based administration of service delivery in health care has, in itself, a great potential to increase efficiency (appointment management, patient notification, patient information, documentation of services, e-prescribing, communication of test results, etc.) [6], but new technologies go beyond simply replacing paper-based with electronic documentation. Telemedicine utilizes advanced communication technologies to enable distance diagnosis, therapy and monitoring, in which the participants of the health procedure are at different geographical locations and connected electronically. Teledentistry is one subgroup of telemedicine defined by the medical specialization, dentistry, and in addition to dental practice, it extends to dental research, education and management, as well [7, 8]. The common to all telemedicine solutions is that they make the data, the information travel instead of the patient, relatives and health workers, with the data being processed real time (e.g. in the case of videoconferencing), or at different time points (store-and-forward systems). Typical teledentistry solutions include tele-consultation, when the patient-doctor encounter is managed through videoconferencing (D2P) and tele-support, when the doctor treating a particular patient receives support from another doctor, who is at a different location (D2D). The technological development has made it possible to perform not just diagnostic, but therapeutic procedures, such as surgical interventions, from a distance, and while the doctor being present with the patient is only for reassurance, the opportunity can also be used for training purposes [7, 9, 10].

According to the available literature, teledentistry solutions have been successfully implemented in Australia, the USA and England [7, 11, 12]. They delivered improved access to services in rural, remote areas and reduced travel for patients and their families. For instance, in Australia, the government has an ongoing struggle to provide access to high quality dental services for the population of remote geographical areas [13]. Although the dental care of school-age children is fully covered, human resources shortages have created large regional disparities manifested in long waiting times [14, 15]. According to a study by the Clinical Department of the University of Melbourne, the introduction of a cloud-based, store-and-forward teledentistry system was able to reduce unnecessary patient visits, waiting times, and could save 275.75 h of work (i.e. 36.7 days) per year, while 95% of the patients was satisfied with the service and found the software user friendly [16]. Further, dentists in rural areas with backward infrastructure and unfavorable working conditions could receive on the job training in the form of telesupport [16].

The experiences suggest that one of the most important advantages of teledentistry is the more efficient use of human resources, which has recently become a critical factor of health systems performance all over the world [17, 18]. The recent COVID-19 pandemic has shown, how even relatively well staffed health systems can easily be overwhelmed with a sudden surge of critical condition patients, and the bottleneck of upscaling meaningful health care capacities is not necessarily the special pieces of equipment needed, but the appropriately trained health care workers to operate them [19]. Digital health solutions have been widely used to support coping with the challenges posed by the COVID-19 pandemic in this respect [20–23]. Hungary also exemplifies the importance of the health workforce, as the country has been experiencing a serious human resources crisis in the health sector, due to the massive emigration of doctors and other health professionals into the higher income countries of the EU, especially Germany, the UK and the Scandinavian countries [24–26]. The shortage is even more worrisome, if we consider that Hungary had a substantial surplus of physicians during the communist era [27].

As far as dentistry is concerned, the situation is not at all better, despite the fact that the number of dental graduates are increasing (Table 1). According to WHO guidelines, no more than 2000 inhabitants should be cared for by 1 dentist. Theoretically Hungary meets this criterion, as on the basis of the number of licensed dentists and the size of the population, there were on average 1708 inhabitants per dentist in 2015. However, if we take into account the number of publicly financed dental practices only, this figure was as high as 3370 [28]. The almost twofold difference is attributable to fact that there is a sizeable private sector in Hungary, which a huge percent of the population cannot afford to utilize.

**Table 1:** The state of human resources in dental care in Hungary, 2015. *Source*: (1)

Indicator	Figure for 2015
Number of inhabitants per dentist	1708
Number of inhabitants per publicly financed dentist	3370
WHO recommendation	2000
Number of vacant practices	280
Number of permanent vacancies	42
Average age of dentists (year)	50.5
Share of dentists over the pension age (%)	20

Further, the averages hide large regional disparities. In terms of dental primary care, there are currently 280 vacant practices, and the number of permanent vacancies (practices, which have been unoccupied for over 10 years) is as high as 42 [28]. The trends are also worsening, despite that universities supply close to 300 new dentists in each year. Fresh graduates are reluctant to work in deprived rural areas and migration also takes its toll. Patients living in these remote locations are forced to travel, sometimes for hours, to central dental providers, which are also responsible for specialist dental care, such as periodontal and orthodontic care, oral medicine or dental surgery, as well as the care for patients with special needs, such as multiple chronic diseases, or disabilities [29]. As a result, central dental providers are jammed, dentists struggle with lack of time, while waiting times are increasing and unacceptably long.

The human resources crisis in dental care is further aggravated by aging. The average age of dentists is 50.5 years, and 20% of dental professionals are over the pension age [28]. Sudden external shocks, such as the COVID-19, further amplify these tensions [30]. In this paper, we have studied how teledentistry could be applied to ease the performance problems created by the shortage of dental health professionals, using the example of a university clinical department (Department of Community Dentistry, Semmelweis University, Budapest), which provides the full spectrum of dental health services from primary to tertiary care.

## Methods

The study is based on a survey of the dental care provided by the Oral Medicine Unit, the Peridontology Unit and the Orthodontics Unit of the Department of Community Dentistry, Semmelweis University, Budapest. The present study investigates dental examination and diagnostic hours comparing the personal examination and teledentistry facilities. The Oral Medicine Unit regular surgery hours are from 8.30–14.00 and from 14.30–20.00 daily, 5 days a week, and it provides tooth preserving and dental prosthetics services and treats diseases of the oral mucous membrane. During a period of 12 weeks, we have collected time balance data on a total of 539 regular patient visits compared to 592 patients, who were attended in a pilot teledentistry setting, in order to estimate the worktime, which could be spared with the roll out of teledentistry technologies to all eligible visits.

The Department of Community Dentistry of the Faculty of Dentistry, Semmelweis University, Budapest, is the only dental care provider in the Hungarian health system, which has exclusively public employees and it has a contract with the National Institute of Health Insurance Fund Management (NEAK) to provide the full spectrum of primary and specialist ambulatory dental care and dental surgery. Hungary has a social health insurance system with a single national pool (the Health Insurance Fund), managed by the NEAK, which contracts mainly with public providers for the provision of health services to the population [31, 32]. The benefit package covers almost every dental and oral prevention service, dental treatment, emergency intervention, periodontal therapy and orthodontic procedure, but co-payment is required for prosthodontic treatment of individuals from 18 to 62 years of age [29, 33]. Each patient also pays the technical costs associated with dental treatment. This is unique in Europe. The service delivery system is organized on the basis of the so-called territorial supply obligation, in accordance of which each service provider is assigned a catchment area, whose inhabitants have to be cared for by this provider, but the same provider can have different catchment areas for different services (usually the smallest catchment area is in primary care—these are called primary care districts, and the largest is the whole country for highly specialized tertiary care services) [31]. The Department of Community Dentistry has to provide primary dental care for the inhabitants of district VIII of Budapest and Budapest, Pest County and Nógrád County is the catchment area of specialist and emergency dental care. In practice, as a university clinical department, it accepts patients from the whole territory of Hungary.

One dental shift is comprised of a number of episodes of care, in the frame of which a patient is attended by the staff of the Department of Community Dentistry in one of its 35 chairs. Each episode of care is by and large divided into an administration period and a treatment period. The administration period is comprised of the patient registration at the coordinator desk and the recording of the visit in the medical information system of the university, together with the scheduling for the next appointment after the completed intervention. The treatment period, by and large, can be divided into either an actual intervention, such as tooth filling or root canal treatment in primary dental care, and tooth extraction in dentoalveolar surgery, or consultation, including for instance patient monitoring, prevention advice, treatment modifications and certain diagnostic procedures, which do not necessary require physical contact with the patient. A substantial part of patient visits, for instance, in specialist dental care, such as orthodontics, periodontology, and oral medicine fall into this latter category.

In the frame of this study, we first identified the type of visits, which can be supported or replaced entirely with teledentistry solutions. Three techniques have been considered. The first is the so-called “store-and-forward”, in which the patient data, such as physiological data, medical notes, various photos and videos are stored electronically and made accessible to another specialist at a distant geographical location. This is already a well-known, and widely applied technique in the evaluation of diagnostic imaging. As opposed to the first technique, the second is real time consultation, such as a video-conferencing session that allows the participants at different geographical locations to exchange information at once, without delay. This system is useful, for instance, for consultation about periodontal treatments or dysgnath surgeries. The third technique is the so-called “remote patient monitoring”, which is a mixture of the two previous options, when biometric data are obtained and stored before the session and made accessible during the session, or generated online, during the session with appropriate equipment [34–37]. These can save time either by shortening of a session, or by replacing a regular face-to-face session with a virtual one. For instance, recall examinations can be provided by using photos of an intraoral camera or smartphone [6, 12]. Consultations, requesting or providing second opinion can also be managed in a telemedicine system.

By considering which types of visits are amenable to teledentistry, we have created 3 categories: prevention, remote patient monitoring and other consultations. In the frame of this study we identified appropriate cases of remote patient monitoring in 3 specialist areas: orthodontics, periodontology and oral medicine. During the data collection period, we recorded the time balance data of 155 scheduled regular visits, with the patients being present physically, in orthodontics, 187 in periodontology and 197 in oral medicine, over a 12-week period in two shifts, and calculated the average time per patient for each specialty. We have done the same with 184 orthodontics visits, 200 periodontology visits and 208 oral medicine visits in a pilot telemedicine setting, where the visits have been managed virtually in the frame of a locally developed application, and compared the data of these two settings.

We have expected that attending the same type of patients in the teledentistry setting does not only good for the actual patients, who spare the travel, registration and waiting time, but the patient-doctor encounter also requires less time due to the more efficient administration of the case (integrated patient history, case documentation, appointment scheduling) and the sparing of time required for the preparation of accepting the next patient (e.g. disinfection procedures). First we calculated the average time difference per remote patient monitoring visit in each specialty and then estimated the total time that could be spared and used to attend more patients, on the basis of the total patient turnover of the Department, regarding the relevant cases in the 3 specialties concerned.

## Results

Table 2 presents the time data of a patient visit, based on the average of altogether 539 patients in the regular setting and 592 patients in the teledentistry setting. All the patients in the regular setting spends a couple of minutes with registering at the coordinator desk, which, of course does not apply to patients in the teledentistry setting. The average of the actual consultation time of remote patient monitoring varies with the specialty. As Table 2 shows, attending a patient in a teledentistry setting instead of the traditional patient-doctor encounter results in some time saving, which varies between 5 to 10 min, orthodontics yielding the least with 5.4 min and periodontology the most with an average of 10.0 min.

**Table 2:** Time utilization in specialist dental care without and with teledentistry (in minutes)

	Avg. consultation time per patient	SD	Ci	Cl min	Cl max	N
*Regular*						
Orthodontics	20, 40	1.71	0.269	20, 10	20.6	155
Periodontology	21, 10	1.50	0.215	20, 90	21.3	187
Oral medicine	29, 80	3.37	0.471	29, 40	30.3	197
*Tele*						
Orthodontics	15, 00	3.58	0.517	14, 50	15.5	184
Periodontology	11, 10	2.11	0.292	10, 80	11.4	200
Oral medicine	20, 30	2.40	0.326	19, 90	20.6	208

If we add up these seemingly small performance improvements, the capacities can be increased and waiting times can be reduced significantly. Calculating with 5 workdays per week, and 46 weeks per year, this improvement is summing up to 58–116 h, which are equivalent to almost 10–20 workdays per doctor, according to specialty (Table 3). Further, should the pilot study be rolled out to all chairs within a shift (i.e. 13 chairs), we are talking about as many as 186 workdays per year and twice as many, i.e. 372, in two shifts. It is important to note that this figure assumes that all the remote patient monitoring visits in the studied 3 specialties can be shifted from the regular face-to-face to the teledentistry setting. In practice, not all the patients are capable of handling a videoconferencing session, which reduces somewhat the time saving that can be achieved realistically.

**Table 3:** Advantages of the deployment of teledentistry solutions in terms of working hours and workdays spared

Estimated work time	Per day (shift)	Per week (5 days)	Per year (46 weeks)	In work days	% Of the total
	In hours	In hours	In hours		
Regular surgery hours per doctor	6.00	30.00	1380.00		
Surgery with teledentistry solutions					
Orthodontics	5.73	28.67	1318.72		
Periodontology	5.50	27.48	1264.12		
Oral medicine	5.75	28.75	1322.56		
Difference					
Orthodontics	0.27	1.33	61.28	10.21	4.4
Periodontology	0.50	2.52	115.88	19.31	8.4
Oral medicine	0.25	1.25	57.44	9.57	4.2
Roll out to all doctors/chairs					
Orthodontics (5)	1.33	6.66	306.42	51.07	4.4
Periodontology (6)	3.02	15.12	695.29	115.88	8.4
Oral medicine (2)	0.50	2.50	114.88	19.15	4.2
Total	4.85	24.27	1116.59	186.15	6.2
Roll out with the involvement of inactive personnel					
Orthodontics (5)	5.05	25.26	1161.81	193.63	16.8
Periodontology (6)	6.36	31.82	1463.60	243.93	17.7
Oral medicine (2)	1.56	7.78	357.83	59.64	13.0
Total	12.97	64.85	2983.24	497.64	16.6

In addition, teledentistry solutions have the potential to mitigate human resource shortages by making it possible to utilize the professional know-how of those chairside doctors, who are temporarily or permanently unable to provide direct medical care. There are many professionals from different levels, who would be able to take part in teledentistry programs. Highly qualified doctors prior to pension or retired dental specialists are such examples. Doctors sustaining sick leave (for example as a result of tumor therapy, trauma, hand issues, or doctors receiving physiotherapy), who are eager to work, but are not able to provide manual interventions, may be involved. Doctors on maternity leave (sometimes with fresh graduation and up-to-date knowledge) could also join the professional side of teledentistry (mostly store-and-forward type as 2–2.5 h can be spent on teledentistry while childcare is provided). In the case of the Department of Community Dentistry we are talking about 6 dentists on maternity leave, which is equivalent to 36–45 work hours weekly, calculating with 3 workdays per dentist per week, i.e. at least 6 full shifts on one “additional chair” weekly.

Further, qualified dental hygienists may enter into a connected practice relationship with a dentist to provide oral health care services for underserved populations without general or direct supervision in public settings [12, 13, 38]. In case all of the potential remote patient monitoring teledentistry cases could be attended by dentists, who are unable to provide direct medical care, or by dental hygienists, whose work roles are enhanced, a total of close to 3000 work hours (and close to 500 workdays) could be freed for the regular staff in one shift alone (Table 3), and twice as many in two shifts, which translate to the care of about 12,000 additional patients in a year. These time savings and more efficient capacity utilization can be increased further with considering prevention and consultation visits, which are also amenable to teledentistry.

## Discussion

### Summary of main findings

Our small scale pilot study of comparing the time required to attend 539 patients in the regular setting and 592 patients in the teledentistry setting at a community dentistry department of a medical university in Budapest, Hungary suggest, that teledentistry could be one of the solutions to the capacity problems of public dental care and to improve access to care for patients in rural areas. The seemingly small time saving of 5–10 min per visit could translate into, as many as, 186 additional workdays per year in the three specialities studied in one shift alone, and almost 500 workdays, if dental hygienists and inactive doctors, who are unable to work in the face-to-face setting, could be involved.

These are important findings, since many rural, remote and outer suburban areas all over the world receive inadequate oral health services, due to workforce shortage in primary care, and the centralization of specialist dental care in urban centers. These patients, that live far from dental centers, have to shoulder the travel costs, as well as the time costs of travel and waiting, even if the services, themselves, are covered publicly.

Hungary is no exception with substantial temporary and permanent vacancies of primary dental care practices in socially disadvantaged regions of the country. Having an extensive private sector for the well-off, the availability of publicly financed dentists is well below the recommendation of WHO, and specialist community dental centers, such as the one at Semmelweis University in Budapest, struggle with a very high patient turnover. Other countries deal with similar issues. In Australia, Dudko et. al. suggest, that the involvement of private dental providers in public dental care could be a reasonable solution [39], but we argue that the problem could also be addressed within the public sector by utilizing the efficiency enhancing potential of the technological development of our age.

Teledentistry with its technologies has an enormous potential to increase the availability of oral health specialists by making the delivery of traditional face-to-face dental care more efficient, and by using information and communication technologies to create new forms of care, which connect care processes among patients, primary care doctors, specialists and other health workers staying in different geographical locations [40]. As an example, patient education and prevention could be introduced throughout video streaming webpages, where medically supervised information could be found about illnesses. Di Stasio et al. found that most of the videos on a specific oral medicine-related problem was uploaded by generalists [41]. Also, photos of oral lesions could be sent to specialists via mobile phone application that can reduce referral decision to special care from 96.9 to 35.1% [42]. It is the dentist, who should consider the type of consultation (tele or face-to-face) that is needed for the patient, in order to achieve the best therapeutical effect [43].

In our pilot project, just by the application of teledentistry solutions in remote patient monitoring, we have shown, that about 1–2 more patients could be treated per doctor per shift, which would add up to 4500 patients more in a year in orthodontics, periodontology and oral medicine. On the other hand, teleconsultation could be another helpful tool, if we would like to raise the time-effectiveness in other areas, as well. Emergency dentistry could benefit from pre-triage systems and teledental consultations [44]. Also, children’s dental screening could be managed by teledental solutions [45].

With teledentistry, we could involve those chairside doctors, who are temporarily or permanently out of direct patient care, or dental hygienists with enhanced competencies to further improve the patient throughput, to 12,000 additional visits per year. These methods could also support the substitute/replacement doctors in rural areas, with remote specialists taking over patient diagnostics and providing consultancy.

Nevertheless, unlike in Australia, where government project funding has allowed the centers to investigate different workforce strategies for increasing access to care, such as telehealth, care coordination programs, mobile technology and financing of graduate medical education [16, 46], currently there is no dedicated funding available to support the implementation of such dental care initiatives in Hungary. The COVID-19 pandemic did give a push to the systemic application of digital health solutions in medicine, such as the widespread application of the e-prescription system, the inclusion of telemedicine services in public financing, and the extension of the functions of the Hungarian eHealth Cloud, but public dental care unfortunately still lags behind [47, 48].

Unfortunately, task shifting to dental hygienists is also problematic in Hungary, since the general regulatory framework does not let the full delegation of medical tasks to qualified health professionals. The same issue plagues other areas, such as the deployment of advanced practice nurses in pain management [49].

### Strengths and limitations

The strength of this study is that it addresses an important problematic issue of community dentistry and demonstrates the potential of teledental solutions in solving the problem of capacity constraints by improving the efficiency of and increasing the access to public dental care. It was conducted by specialists, who attend dental patients daily in the public sector.

The main limitation of the pilot is that it has been confined to only one center in one country, Hungary, with a relatively small number of participants. A multi-center research would be useful in the future to be able to compare the differences between centers, regions and countries, as well as different settings and levels of care. Further, we investigated the use of teledentistry only from the point of view of time-effectiveness. Other aspects, such as the reduction of face-to-face visits, the costs of implementation and other dimensions of feasibility should also be considered in future studies.

### Future directions

In addition to an extended study with a broader scale and scope in community dentistry, the application of digital health solutions in other areas of dental care should also be explored and studied. Primary dental practice, gate-keeping by assisted pre-triage, and graduate and postgraduate education are a few examples, where teledentistry can also prove to be useful, but have not been addressed in Hungary, yet.

## Conclusions

Our case study has shown that teledentistry has a great potential to offer better access to dental care, both by improving the time utilization of traditional dental care, and as an alternative to classic face-to-face dentistry, but the socially indigents will unlikely benefit from these opportunities without a systematic development program of the digital renewal of dental care. According to the investigation, the usage of teledentistry is recommended. Hungary desperately needs such a policy change as public dental care struggles with capacity problems, whose solution will unlikely be forced by the wealthier part of the society, since they have already left the public sector, and utilize dental services with the providers of a well-developed private sector.

## Data Availability

The datasets generated and analysed during the current study are not publicly available due to the impossibility of earning the datas anonimly. In proper measuring examiners it is not possible to keep the anonimity of the patients, but are available from the corresponding author on reasonable request.

## Abbreviations

ICT: Information and communication technologies
D2P: Doctor to patient
D2D: Doctor to doctor
WHO: World health organization
NEAK: National Institute of Health Insurance Fund of Hungary
